# 聚氯乙烯微塑料对典型单羟基菲的吸附机制

**DOI:** 10.3724/SP.J.1123.2020.09005

**Published:** 2021-08-08

**Authors:** Zhenzong BAO, Zhifeng CHEN, Zenghua QI, Guangzhao WANG, Zongwei CAI

**Affiliations:** 1.广东工业大学环境科学与工程学院, 广东 广州 510006; 1. School of Environmental Science and Engineering, Guangdong University of Technology, Guangzhou 510006, China; 2.香港浸会大学化学系, 环境与生物分析国家重点实验室, 香港 00852; 2. State Key Laboratory of Environmental and Biological Analysis, Department of Chemistry, Hong Kong Baptist University, Hong Kong 00852, China; 3.长江师范学院电子信息工程学院, 超常配位键工程与新材料技术重庆市重点实验室, 重庆 408100; 3. Key Laboratory of Extraordinary Bond Engineering and Advanced Materials Technology of Chongqing, School of Electronic Information Engineering, Yangtze Normal University, Chongqing 408100, China

**Keywords:** 聚氯乙烯, 3-羟基菲, 紫外分光光度计, 吸附, 机理, polyvinyl chloride, 3-hydroxyphenanthrene, ultraviolet spectrophotometry (UV), adsorption, mechanism

## Abstract

为丰富微塑料与有机污染物间的相互作用机制相关数据,以3-羟基菲(3-OHP, C_14_H_10_O)为菲单羟基衍生物代表污染物,聚氯乙烯(PVC)微塑料为研究对象,研究了PVC微塑料在水环境中对3-OHP的吸附行为,并就相关吸附机制进行了深入探讨。该研究借助扫描电镜(SEM)、X射线衍射仪(XRD)、傅里叶红外光谱(FT-IR)等仪器对PVC微塑料进行表征,利用紫外分光光度计得出目标污染物的紫外吸收光谱标准曲线,标准曲线拟合相关系数(*R*^2^)>0.99。为保证紫外吸收光谱的准确性,污染物浓度梯度设置为吸光度(Abs)大于0.438,之后根据标准曲线方程计算其浓度,结合相关吸附模型(吸附动力学、吸附等温线和吸附热力学)并配合密度泛函理论(density functional theory, DFT)探讨了在水环境中PVC微塑料对3-OHP的吸附机制。结果如下:(1)吸附动力学实验结果显示伪二级动力学模型拟合程度最好,吸附动力学拟合系数*R*^2^=0.998。因此,PVC吸附3-OHP可能是以表面吸附和外液膜扩散的吸附方式,吸附发生24 h后的平衡吸附量为36.866 μg/g; (2)吸附等温线实验表明Langmuir和Freundlich等温线模型拟合度较高,吸附等温线拟合系数*R* ^2^分别为0.956和0.907,更加适合描述PVC对3-OHP的吸附过程,吸附模式主要为单层吸附,也存在小部分多层吸附,PVC对3-OHP的最大吸附量为408 μg/g; (3)吸附热力学结果显示PVC微塑料对3-OHP的吸附效率随着温度升高而降低,这表明PVC对3-OHP的吸附为自发、放热的吸附反应;(4)盐度实验结果表明,盐度对3-OHP在PVC上的吸附效率影响不大;(5)DFT理论计算结果表明PVC对3-OHP结合能相对较低,因此推测PVC对3-OHP的主要吸附机制可能是疏水作用,还可能存在弱氢键作用、卤素键作用以及*π-π*共轭作用。研究揭示了PVC微塑料与有机物相互作用方式,明确了PVC微塑料对3-OHP的吸附模式,探讨了PVC微塑料对3-OHP的相互作用机制,有助于更好地了解PVC微塑料在水溶液中的环境行为。该研究为科学评价微塑料的环境影响提供数据参考,并进一步补充了微塑料的毒理学机制数据。

## Abstract (en)

To enrich data related to the interaction mechanism between microplastics and organic pollutants, in this study, 3-hydroxy-phenanthrene (3-OHP, C_14_H_10_O), a phenanthrene derivative, was selected as a representative pollutant, and polyvinyl chloride (PVC) microplastics were chosen as the research objects. We investigated the adsorption behavior of 3-OHP on PVC microplastics in aqueous solutions and explored the adsorption mechanism in detail. The PVC microplastics were characterized by scanning electron microscopy (SEM), X-ray diffraction (XRD), and Fourier transform infrared (FT-IR) spectroscopy. The standard curves of the ultraviolet (UV) absorption spectrum of the target pollutant were obtained using a UV spectrophotometer. The fitting coefficient values of all standard curves were higher than 0.99 (*R*^2^>0.99). To ensure the accuracy of the UV absorption spectrum, the pollutant concentration gradient was set according to the absorbance (Abs) values, which were higher than 0.438. The measured concentrations were calculated using a standard curve equation. The adsorption mechanism of 3-OHP on PVC microplastics in an aqueous solution was studied by combining adsorption models (adsorption kinetics model, adsorption isotherm model, and adsorption thermodynamics model) and density functional theory (DFT) calculations. The results are as follows: (1) From the adsorption kinetics experiment, the pseudo-second-order kinetic model had the best fitting degree, and the fitting coefficient of adsorption kinetics was 0.998 (*R*^2^=0.998). Hence, 3-OHP adsorption on PVC microplastics may be attributed to surface adsorption and external liquid film diffusion; the equilibrium adsorption amount was 36.866 μg/g after 24 h. (2) The adsorption isotherm experiment showed that the Langmuir and Freundlich isotherm models were more suitable for describing the adsorption mechanism of 3-OHP adsorption on PVC microplastics because of the satisfactory fitting coefficient (*R* ^2^=0.956 and 0.907), suggesting that the adsorption mode was mainly single-layer adsorption with a small amount of multilayer adsorption. The maximum adsorption amount of 3-OHP adsorption on PVC microplastics was 408 μg/g; (3) the adsorption thermodynamics results showed that the adsorption efficiency of 3-OHP adsorption on PVC microplastics decreased with increasing temperature, indicating that the adsorption of 3-OHP on PVC microplastics was a spontaneous and exothermic adsorption process; (4) the salinity experiment results showed that salinity had little effect on the adsorption efficiency of 3-OHP on PVC microplastics; (5) DFT calculations showed that PVC had a relatively low binding energy to 3-OHP. Therefore, we suggest that the main adsorption mechanism of 3-OHP on PVC microplastics may be the hydrophobic effect; weak hydrogen bonding, halogen bonding, and *π-π* conjugate action could also play a role in 3-OHP adsorption on PVC. These results reveal the interaction mechanism between PVC microplastics and organic chemicals, and enhance our understanding of the environmental behavior of PVC microplastics in aqueous solutions. To serve as a reference in scientific evaluations of the environmental impact of microplastics, future studies should focus on obtaining toxicological data for the microplastics.

目前,塑料废弃物的不可持续性处理是地表水和海洋的一个新兴环境问题^[1,2,3]^。据估计,每年有超过3.2亿吨塑料被丢弃在自然环境中^[4]^,约10%的塑料通过各种方式进入水环境中^[5]^。有报道指出塑料颗粒量约占海洋垃圾总量的60%~80%^[6]^,随着洋流运动广泛分布于全世界的海洋环境中^[7]^。微塑料通常被定义为直径小于5 mm的塑料微球。环境中的微塑料依据来源分为初生微塑料和次生微塑料两大类。初生微塑料是直接添加到化妆品、护理产品中小于5 mm的微珠。次生微塑料指大片塑料片经过物理、化学作用被破碎为粒径小于5 mm的塑料粒^[8]^。目前微塑料在湖泊^[9,10]^、河口^[11]^、海洋^[12]^甚至偏远地区被广泛发现^[13]^。在亚洲,微塑料也被发现存在于蒙古库苏古尔湖和中国青藏高原湖泊^[14,15]^。中国武汉城市湖泊群以及第三大湖—太湖也被监测到微塑料污染^[16,17]^。此外,由于尺寸较小,微塑料可能会被海洋生物摄取^[18]^,作为污染物的载体进入生物体,并可能对生物体造成健康风险^[19]^。有研究证实在海洋中层以浮游生物为食的35%鱼类内脏中检测出微塑料^[20]^。Murray等^[21]^研究表明,83%的鳌虾都会吞食微塑料,并且在体内富集。Brillant等^[22]^的室内模拟实验研究发现,浮游动物和海洋生物均会摄取微塑料。目前,针对微塑料的研究集中在微塑料的时空分布及其对水生生物毒理机制研究方面,而对微塑料与有机污染物相互作用机制研究较为缺乏。

微塑料因具有疏水性,且带有不同基团等,容易吸附有机污染物、重金属等有害物质^[23]^,如多环芳烃(PAHs)^[24]^、多氯联苯(PCBs)^[25]^、滴滴涕(DDT)^[26]^、多溴联苯醚(PBDEs)^[27]^和重金属等^[28]^。

多环芳烃及其衍生物的环境行为近来已成为一个备受关注的环境问题。PAHs是环境中较为常见的持久性有机污染物之一,具有致癌和诱变特性^[29]^,它们主要通过生物质燃烧、汽车尾气和工业活动产生^[30]^。在大多数自然水域,菲的质量浓度经常为pg/L~ng/L水平^[31]^,而在一些污染严重的水域,如石油废水,菲的浓度可能高达7.6~9.9 μg/L^[32]^,而微塑料极有可能成为其迁移转化的重要载体之一。目前针对多环芳烃在大气中的时空分布特征研究较多,而多环芳烃在水中的环境行为研究较为缺乏。

因此,本研究以聚氯乙烯(PVC)微塑料及典型多环芳烃菲单羟基衍生物(3-羟基菲)为研究对象,通过室内模拟实验和相关模型探究羟基多环芳烃在微塑料上的吸附规律,明确微塑料与羟基多环芳烃的吸附特性,同时基于密度泛函数理论计算出微塑料与羟基多环芳烃的吸附能,进一步阐明微塑料与羟基多环芳烃的界面相互作用方式,以了解水环境中微塑料与疏水性有机污染物的相互作用机制,同时为科学合理地评价微塑料的生态环境风险和相关研究提供重要依据。

## 1 实验部分

### 1.1 仪器、试剂与材料

X射线衍射仪(XRD, D8 ADVANCE,德国);场发射扫描电镜(SEM, S4800,日立,日本);傅里叶变换红外光谱(FT-IR, Thermo-Fisher,美国);紫外分光光度计(MAPADA UV-3200,中国); 纯水仪(NIKO水净化系统,重庆);恒温振荡器(SHA-BA,常州)。

3-羟基菲(3-OHP, 10 mg)购自上海阿拉丁生化科技股份有限公司,纯度为98%。色谱级甲醇购于上海安谱实验科技股份有限公司。PVC微塑料(CAS: 9002-86-2)购自东莞华创塑料化工有限公司,室温干燥保存,颗粒平均粒径为150 μm。

### 1.2 标准曲线绘制

用色谱纯甲醇将3-羟基菲预溶为50 mg/L母液,之后用超纯水稀释至0.05、0.1、0.2、0.5、1、1.5、2.5 mg/L,通过紫外分光光度计测出吸光度,得出3-羟基菲溶液标准曲线方程为*y*=0.16*x*+0.0065,相关系数(*R*^2^)为0.991。

### 1.3 吸附试验设计

为防止光照和蒸发导致的实验误差,在250 mL带旋塞盖棕色锥形瓶中对PVC进行了3-OHP的间歇吸附实验。微塑料表面的化学残留物或有机溶剂可能会影响疏水性有机污染物在微塑料上的吸附,因此对PVC微塑料进行充分的洗涤,用冷冻干燥器干燥后保存在干燥器内备用。

吸附等温线试验:温度25 ℃,振动速度为150 r/min,吸附时间60 h,PVC浓度为15 g/L, 3-OHP初始质量浓度为0.2、0.5、1、1.5、2.5 mg/L。

吸附动力学试验:温度25 ℃,振动速度为150 r/min,吸附时间60 h,PVC浓度为15 g/L, 3-OHP初始质量浓度为1.5 mg/L,采样时间0、0.5、1、3、6、12、24、36、48、和60 h。

吸附热力学实验:温度25 ℃(298 K), 35 ℃(308 K)和45 ℃(318 K),采样时间24 h,3-OHP初始质量浓度为1.5 mg/L。

### 1.4 理论计算

在本研究中,密度泛函理论(DFT)作为一个强大的工具,被用于探索PVC和3-OHP之间的相互作用^[33,34]^。采用Vienna Ab-initio Simulation Package (VASP )进行理论计算^[35]^。分别对PVC、3-OHP和PVC+3-OHP进行模型优化,然后将优化后的PVC、3-OHP和PVC+3-OHP模型按最佳组合条件进行优化计算。最后,结合能(Δ*E*)可以通过下列公式计算:


(1) Δ*E*=*E*_PVC+3-OHP_-*E*_PVC_-*E*_3-OHP_

其中Δ*E*为结合能,结合能值越低,说明PVC与吸附物之间的相互作用力越强。

## 2 结果与讨论

### 2.1 微塑料表征

为了解PVC微塑料的特性,并进一步鉴定PVC微塑料,采用SEM、XRD、FT-IR等进行了表征。图1中a、b为PVC微塑料的显微镜图像。SEM显示出PVC微塑料表面粗糙且形状不规则,具有一定的孔隙结构,系较多褶皱形成的狭长形气孔,此结果与Dong等^[36]^的研究类似。塑料颗粒具有疏水表面,因此,非极性有机物质可以吸附和扩散到聚合物表面或内部。图1c中XRD谱图显示了聚合物PVC结晶的程度,可以看出PVC的结晶度较差,这与Liu等^[37]^的研究结果类似。图1d 清晰地显示出PVC微塑料在红外图谱中的主要特征峰:3448 cm^-1^处的O-H伸缩振动,2915 cm^-1^处的-CH_2_-对称伸缩振动,1648 cm^-1^处的C=C伸缩振动,1434 cm^-1^处的Cl-CH_2_变形振动,1254 cm^-1^处的Cl-CH平面外变形振动,1098 cm^-1^处的C-C伸缩振动,在691 cm^-1^处的C-Cl伸缩振动^[38]^。同时图1d中FT-IR图谱对比结果可知,PVC微塑料吸附3-OHP前后,PVC微塑料红外图谱中主要特征峰未发生增减变化,说明PVC微塑料与3-OHP相互作用力可能是弱的相互作用而非化学过程。

**图1:**
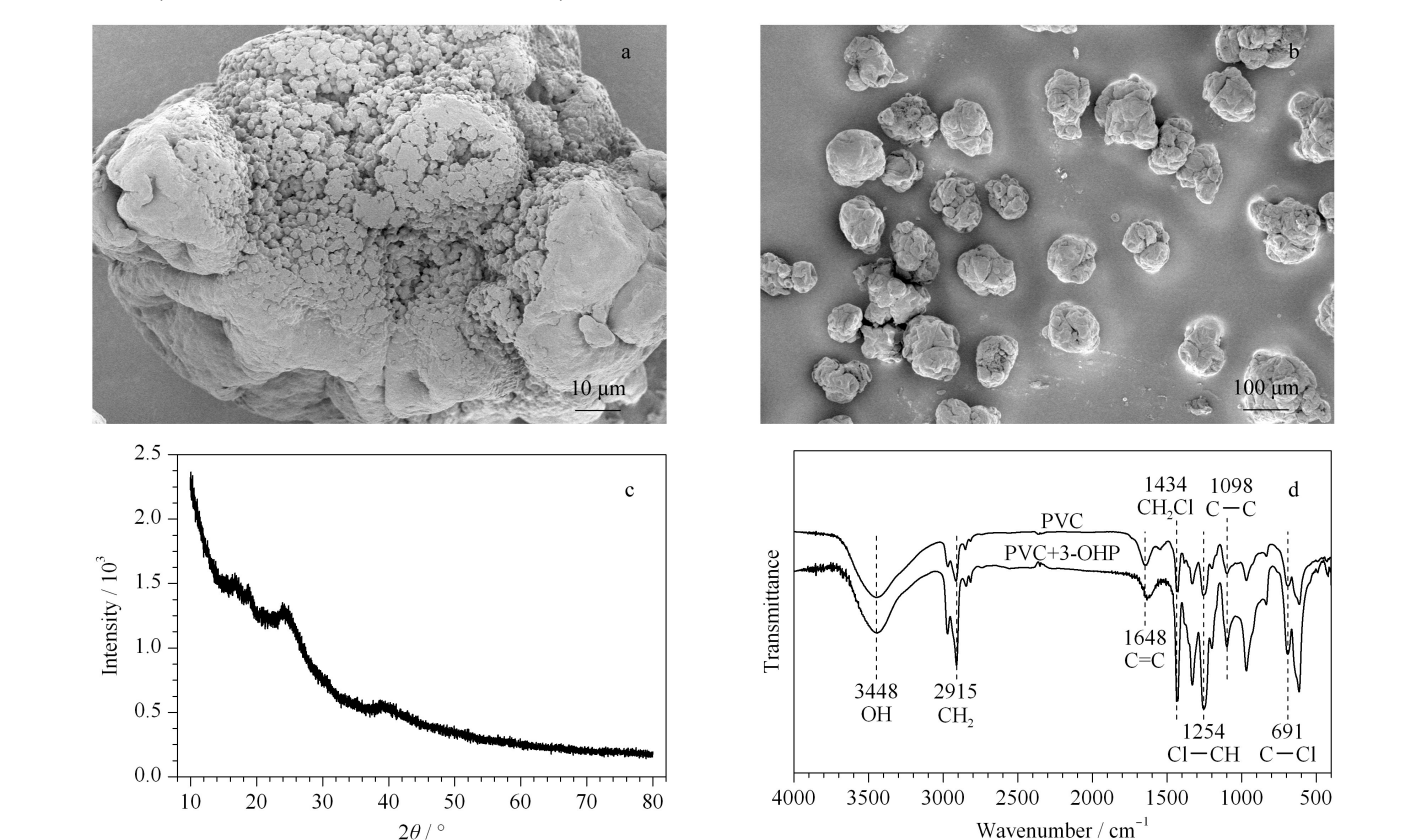
聚氯乙烯(PVC)微塑料表征

### 2.2 吸附等温线

吸附等温线方程通常用于说明吸附物与吸附剂在平衡状态下的相互作用。采用Langmuir、Freundlich、Temkin和Dubinin-Radushkevich模型^[39,40]^拟合了3-OHP在PVC微塑料上的吸附实验数据,得到的参数见表1。Langmuir模型表示吸附过程发生在吸附剂的单一表面,属于单层吸附。Freundlich模型表明PVC微塑料与污染物相互作用之间存在多层吸附,为受化学和化学物质影响的非均匀表面上物理吸附过程^[41]^。Temkin模型可以解释吸附剂与微塑料之间的相互作用和吸附势。Dubinin-Radushkevich模型可以通过估计吸附自由能来判断吸附过程的物理化学性质^[42]^。表1结果表明,PVC微塑料对3-OHP的吸附实验数据在Langmuir、Freundlich模型中拟合度较好,而Langmuir模型中*R*^2^=0.956大于Freundlich模型*R*^2^=0.907,表明PVC微塑料上对3-OHP的吸附作用之间存在多层吸附,但以单层吸附为主。这与Bakir等^[43]^报道的PVC对菲(phenanthrene)和4,4'-滴滴涕(4,4'-DDT )的吸附结果相似。

**表1:** PVC微塑料与3-OHP吸附等温线拟合参数

Langmuir				Freundlich				Temkin						Dubinin-Radushkevich				
*R* ^2^	*K*_L_	*q*_m_/(mg/g)		*R* ^2^	*q*_m_/(mg/g)	*β*		*ε*	*B*	*a*_T_	*b*_T_	*n*		*R* ^2^		*q*_m_/(mg/g)	*β*	*ε*
0.956	3×10^-4^	0.408		0.907	0.094	4.89×10^-4^		211.838	0.826	0.057	2999	1.21		0.871		0.094	4.89×10^-4^	211.838

*q*_m_ is the maximum adsorption capacity (mg/g) of microplastics under monolayer adsorption; *K*_L_ is the surface adsorption equilibrium (Langmuir) constant (L/mg); *a*_T_ and *b*_T_ are the Temkin isotherm constant (L/mg) and Temkin constant (J/mol) related to adsorption heat; *β* is the Dubinin-Radushkevich model constant (mol^2^/J^2^) related to adsorption energy; *ε* is the Polanyi potential.

### 2.3 吸附动力学

为了解PVC微塑料对3-OHP的吸附平衡时间,进行了吸附动力学研究。3-OHP在PVC微塑料上的吸附过程如图2所示:采用4种常用模型分析了3-OHP在PVC微塑料吸附的动力学特性^[7,43,44]^, 3-OHP迅速吸附在PVC微塑料表面,随后扩散到PVC微塑料层间结构的孔隙中。4种动力学模型拟合结果见表2,可以看出,伪二阶动力学模型的拟合相关系数*R*^2^=0.998明显高于伪一阶动力学模型*R*^2^=0.917、内扩散模型*R*^2^=0.791和液膜扩散模型*R*^2^=0.602。伪一阶动力学模型平衡吸附量*q*_e_计算值与实验值相差较大,而伪二阶动力学模型平衡吸附量*q*_e_计算值与实验值吻合度较好,分别为*q*_e,exp_=36.866 μg/g, *q*_e,cal_=37.764 μg/g。吸附动力学数据与伪二阶模型拟合相关系数较高(*R*^2^=0.998),说明3-OHP可以吸附在微塑料的不同结合位点上^[45,46]^。

**图2:**
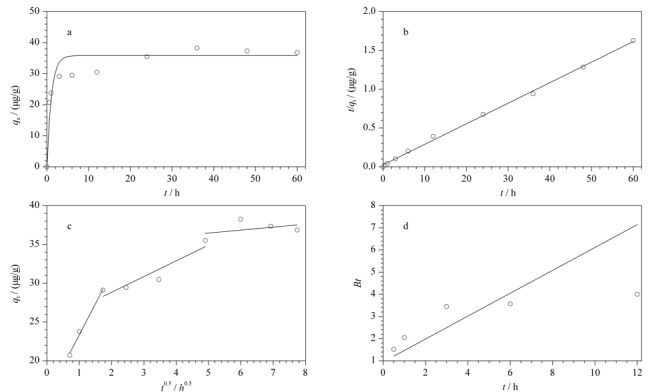
PVC微塑料与3-OHP的吸附动力学

**表2:** PVC微塑料与3-OHP的吸附动力学拟合参数

Kinetics model	*q*_e,exp_ /(μg/g)	equation	*R* ^2^	*k*	*q*_e,cal_/(μg/g)
Pseudo-first order	36.866	*y*=34.336*×*(1-0.238*x*)	0.917	1.436 g/(mg·min)	34.336
Pseudo-second order	36.866	*y*=26.538*x*+22.19	0.998	0.0279 g/(mg·min)	37.764
Intra-particle diffusion		*y*=2.140*x*+23.311	0.791	2.140 mg/(g·min^0.5^)	
Liquid film diffusion		*y*=0.190*x*+2.060	0.602	-	

*q*_e,exp_: experimental equilibrium adsorption quantity; *q*_e,cal_: calculated equilibrium adsorption quantity; *k*: pseudo-first order, pseudo-second order, and intra-particle diffusion equilibrium rate constants, respectively.

为了进一步明确3-OHP在PVC微塑料上的吸附机制,采用颗粒内扩散模型对吸附动力学数据进行了拟合。根据颗粒内扩散模型可知,本研究中吸附过程可分为3个阶段,吸附过程开始的前10 h内为快速吸附阶段,然后10~24 h为缓慢吸附阶段,最后24 h吸附速率达到吸附平衡,吸附动力学实验持续到60 h,直至吸附效率基本无变化。快速吸附阶段为表面的多相吸附,即有机污染物通过疏水作用、共价键和范德华力等附着在微塑料表面;缓慢吸附阶段为外液膜扩散,有机污染物缓慢地从外液膜扩散到了微孔内;最后达到吸附平衡^[44]^。这说明PVC微塑料对3-OHP的主要吸附机制为表面吸附和外液膜扩散。

### 2.4 吸附热力学

从图3中所示的不同温度(298、308、318 K)下的吸附结果可以看出,随着温度的升高,PVC微塑料对3-OHP的吸附效率呈下降趋势。热力学相关参数如表3所示:Δ*G*、Δ*S*、Δ*H*为吉布斯自由能(kJ/mol)、熵(kJ/mol)、焓(kJ/mol); *k*_c_为平衡常数(L/g)^[47]^。

**图3:**
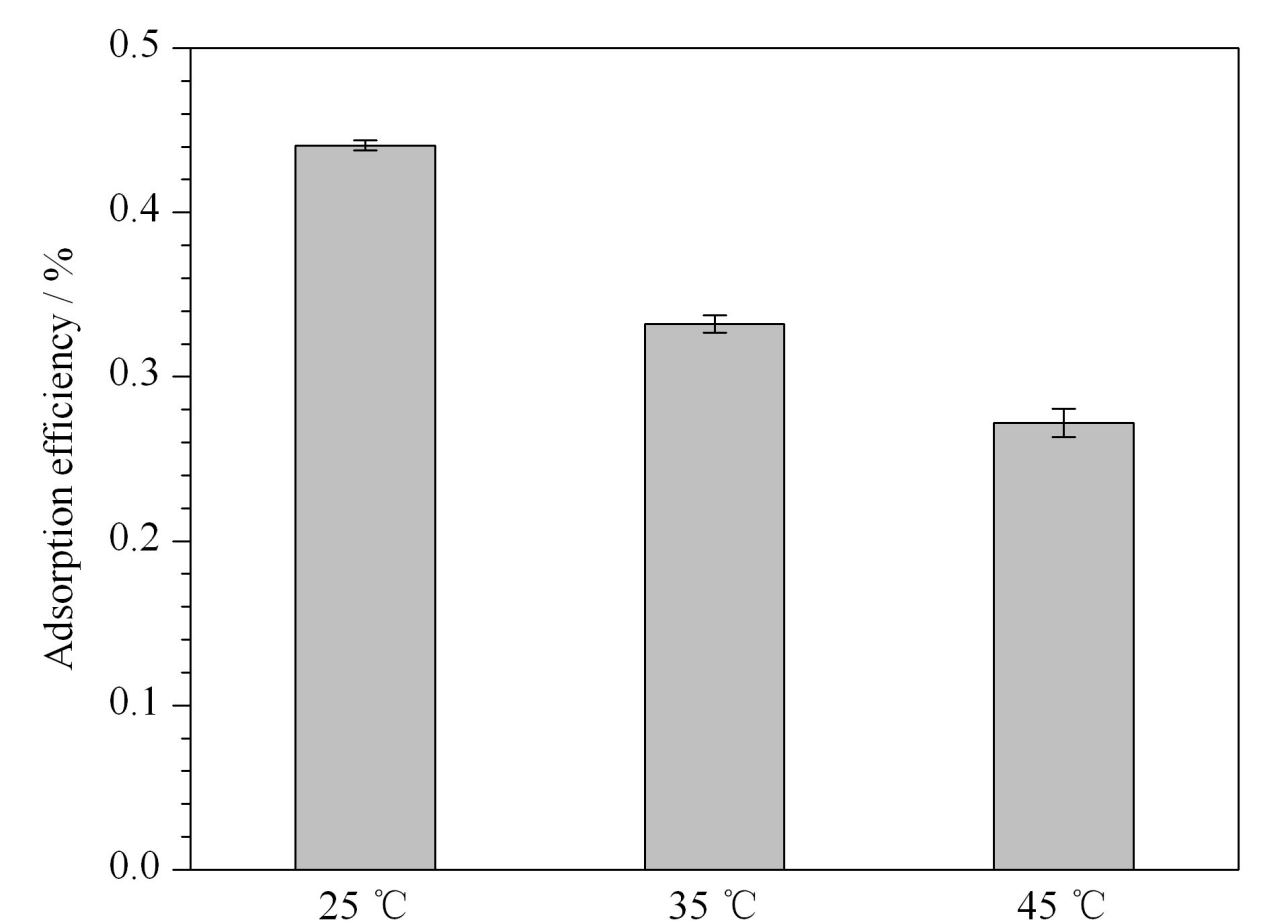
PVC微塑料与3-OHP的吸附热力学(*n*=3)

**表 3:** PVC微塑料与3-OHP吸附热力学拟合参数

ln *k*_c_				Δ*G*/(kJ/mol)			Δ*H*/ (kJ/mol)	Δ*S*/ (kJ/mol)
25 ℃	35 ℃	45 ℃		25 ℃	35 ℃	45 ℃		
4.389	4.426	4.611		-10.875	-11.334	-12.192	-10.15	-0.65831

*k*_c_: equilibrium constant.

进一步研究了PVC微塑料对3-OHP吸附过程中的吉布斯自由能变化(Δ*G*)、焓变化(Δ*H*)和熵变化(Δ*S*)。由表3可知,Δ*H*为负值,表示PVC微塑料吸附3-OHP是放热过程。Δ*G*也为负值,表示在298、308、318 K下PVC微塑料吸附3-OHP是自发过程。PVC微塑料对3-OHP的吸附效率随着温度升高而降低,说明在较高的温度下抑制了微塑料的吸附过程,原因可能是当溶液温度升高时,氢键会断裂,导致吸附反应减弱^[48,49]^。

### 2.5 微塑料对3-OHP的吸附机制

在水环境中,持久性有机污染物可以附着在微塑料表面并不断的迁移。但不同污染物对不同材质的微塑料的吸附方式及吸附量差异较大^[50,51,52]^。本研究中通过图4a可知,在PVC微塑料上吸附多环芳烃类物质时,主要可能是疏水相互作用^[53]^、静电力^[54]^和非共价相互作用^[55]^几种机制。本研究中通过图4b可知,在不同的盐度下,PVC微塑料对3-OHP的吸附效率基本一致,这表明PVC微塑料上吸附3-OHP主要吸附机制可能是疏水相互作用及非共价相互作用。有研究发现疏水分配作用是影响微塑料与有机污染物相互作用的主要机制^[7,56]^,但非共价键如氢键和卤素键也会影响吸附剂与吸附质之间的亲和力^[57,58,59]^。Yamate等^[60]^研究也发现,含苯环的有机物与塑料聚合物之间可能会产生一种弱氢键的CH/*π*相互作用。此外,苯环与苯环之间的*π-π*共轭作用^[7]^、卤素原子与苯环之间的卤键作用^[33]^,也会影响微塑料对有机污染物的吸附。有研究指出^[61,62]^,微塑料对水中污染物的吸附存在吉布斯自由能降低、焓变及熵变小于零的现象,水环境中的有机污染物可由无规则运动向微塑料表面聚集,从而慢慢趋于集中,即为典型的自发、放热反应,这与本文中的研究结果类似(见图3和表3)。

**图4:**
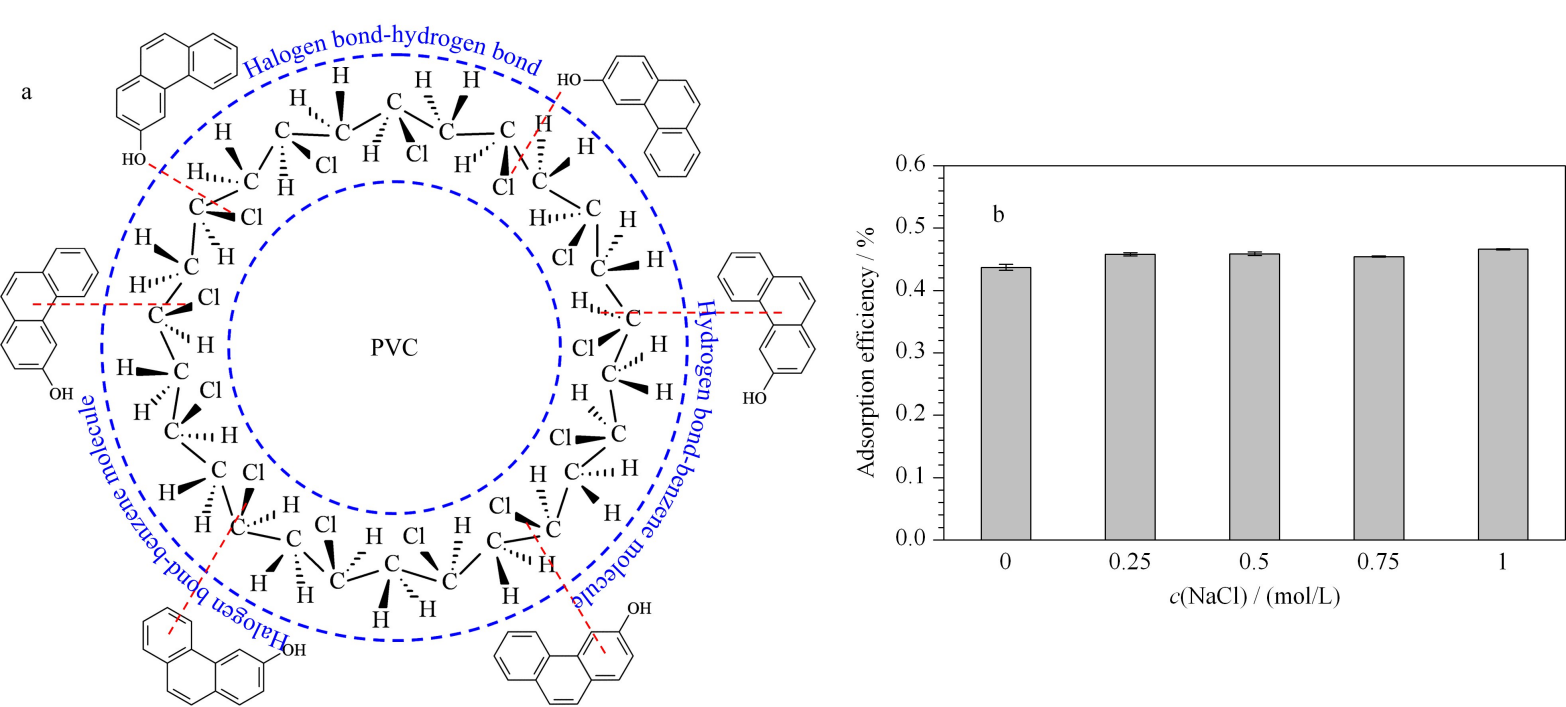
PVC微塑料与3-OHP的吸附机理图

利用密度泛函理论(DFT)对聚氯乙烯微塑料的氢键和卤素键作用进行了计算研究。将优化后的PVC与3-OHP模型按最佳组合条件组装成复合物,在优化组合计算的基础上,得出PVC、3-OHP及PVC+3-OHP复合物的总DFT能(见表4),进而根据公式(1)算出PVC对3-OHP的结合能,一般认为结合能越低,亲和力越稳定。然而,菲的单羟基取代位置不同,吸附效率也不同,同时羟基取代作用的机理尚不明确,需要在今后的研究中进一步阐明。

**表4:** PVC微塑料与3-OHP的理论计算参数

Molecule	Total DFT energy (DFT-E)/eV	Binding energy (Δ*E*)/eV
PVC	-165.692	
3-OHP	-170.417	
PVC+3-OHP	-336.584	-0.475

## 3 结论

吸附动力学表明,3-OHP在PVC微塑料上的吸附均符合伪二阶动力学模型,吸附等温线Langmuir和Freundlich模型的拟合相关性*R*^2^均在0.9以上,表明吸附过程以单层吸附为主,但也存在多层吸附。吸附热力学表明PVC对3-OHP为典型的自发、放热反应。盐度对PVC吸附3-OHP的吸附效率影响不大,表明PVC对3-OHP的主要吸附机制可能是疏水相互作用,还可能存在弱氢键、卤键作用以及*π*-*π*共轭作用。通过理论计算结果可知,PVC对3-OHP结合能相对较低。
